# Dynamic Mechanical Properties of Several High-Performance Single Fibers

**DOI:** 10.3390/ma14133574

**Published:** 2021-06-25

**Authors:** Xudong Lei, Kailu Xiao, Xianqian Wu, Chenguang Huang

**Affiliations:** 1 Institute of Mechanics, Chinese Academy of Sciences, Beijing 100190, China; leixudong@imech.ac.cn (X.L.); xiaokailu@imech.ac.cn (K.X.); 2 School of Engineering Science, University of Chinese Academy of Sciences, Beijing 100049, China; huangcg@imech.ac.cn; 3 Hefei Institutes of Physical Science, Chinese Academy of Sciences, Heifei 230031, China

**Keywords:** single fiber, mini-SHTB, SEM, Weibull distribution

## Abstract

High-performance fiber-reinforced composites (FRCs) are widely used in bulletproof structures, in which the mechanical properties of the single fibers play a crucial role in ballistic resistance. In this paper, the quasi-static and dynamic mechanical properties of three commonly used fibers, single aramid III, polyimide (PI), and poly-p-phenylenebenzobisoxazole (PBO) fibers are measured by a small-scale tensile testing machine and mini-split Hopkinson tension bar (mini-SHTB), respectively. The results show that the PBO fiber is superior to the other two fibers in terms of strength and elongation. Both the PBO and aramid III fibers exhibit an obvious strain-rate strengthening effect, while the tensile strength of the PI fiber increases initially, then decreases with the increase in strain rate. In addition, the PBO and aramid III fibers show ductile-to-brittle transition with increasing strain rate, and the PI fiber possesses plasticity in the employed strain rate range. Under a high strain rate, a noticeable radial splitting and fibrillation is observed for the PBO fiber, which can explain the strain-rate strengthening effect. Moreover, the large dispersion of the strength at the same strain rate is observed for all the single fibers, and it increases with increasing strain rate, which can be ascribed to the defects in the fibers. Considering the effect of strain rate, only the PBO fiber follows the Weibull distribution, suggesting that the hypothesis of Weibull distribution for single fibers needs to be revisited.

## 1. Introduction

High-performance fibers are widely used in aerospace engineering [1], civil construction [2], marine engineering [3], electron components [4], and biomedicine [5] due to their superior mechanical properties such as high specific strength and modulus. Especially, the lightweight and extraordinary high-strength fibers are required in the human impact protection field to fabricate high-performance helmets and body armors.

Some high-performance fibers have attracted much attention in recent decades for their excellent mechanical properties [6,7]. Among them, polyimide (PI), poly-p-phenylenebenzobisoxazole (PBO), and aramid III fibers are regarded as the most promising fibers for body protective systems. Many researchers have focused on the structural advantages and mechanical performance of these three fibers. PI fiber is composed of a large number of rigid conjugated structures such as imide rings and aromatic heterocyclic rings. This unique chemical structure not only possesses large bond energy, but also considerably improves the intermolecular force between the macromolecular chains through π-π interaction, rendering materials with good mechanical properties [8]. According to previous reports [9,10], the tensile strength of domestic PI fiber can reach 3.5 GPa and the modulus exceeds 140 GPa. PBO fiber is one of the most promising members of the polyamide family containing heterocyclic aromatics, and is known as the super fiber of the 21st century. The tensile strength of PBO fiber can reach up to 5.8 GPa, and the elasticity modulus is 270 GPa [11]. Aramid fiber is a liquid crystal polymer composed of long, rigid molecules, with high strength, modulus, and temperature resistance due to the strong orientation of molecular chains along the length direction and the strong inter-chain binding force. One of the aramid fibers, Kevlar-49, possesses a tensile strength of ~2.7 GPa and an elasticity modulus of ~112 GPa, which are superior to those of glass fibers [12]. The strength of aramid III fibers can reach up to 4.5 GPa, implying that aramid III fiber is inferior to PBO fiber in strength.

It is important to determine the dynamic behavior of high-performance fiber composites and understand the dynamic energy dissipation mechanism for high-performance protective structure design. There is abundant literature that addresses the mechanical behavior of a variety of high-performance fiber bundles and fiber-reinforced composites (FRCs) under different loading conditions [13,14,15]. Sun et al. [16] studied the effect of strain rate on the uniaxial tensile behavior of 3D braided E-glass/epoxy composites. Their results showed that the failure strength of FRC increases significantly with the increase in strain rate. The dynamic mechanical properties of Kevlar 49 fiber bundles were studied using a rotating bar impact tensile test device [17]. The strain rate sensitivity of the fiber bundle increases initially and then decreases with the increase in strain rate. Zhu et al. [18] studied the response of Twaron fiber bundles at different strain rates. The results showed the failure stress of the material increased significantly with increasing strain rate.

However, it is not enough to solely research the FRC structure or fiber bundle. In a whole system of fiber-reinforced composite plates, the single fiber is crucial because it is the most basic macro unit and determines the upper strength limit of the structure. The mechanical behavior of single fibers not only reflects the essential performance but also provides a reference for bottom-up structural design. Chen et al. [19] measured the transverse compressive properties of a single Kevlar fiber. The results showed there is a considerable residual strain in the fiber under compression loading, leading to significant energy absorption. Few studies have focused on the dynamic tests of single fibers due to the difficulty of accurately capturing the force of a single fiber. Lim et al. [20] explored the failure strength of an A265 high-performance single fiber, a type of aramid fiber, under quasi-static and dynamic conditions. The results indicated the strength at a strain rate of 1000 s^−1^ is 16.6% higher than that at 10^−3^ s^−1^. Wang et al. [21] studied the dynamic mechanical properties of single fibers using a small free-falling split Hopkinson tension bar (SHTB). The results showed that the strength of a single carbon nanotube (CNT) fiber increases as the strain rate increases, while there is no noticeable strain rate effect for polyacrylonitrile-based carbon fiber. This research indicates that the strain-rate sensitivities of single fibers are diverse due to their intricate structures. Therefore, it is necessary to investigate the strain rate effect of single fibers and understand the related mechanisms during dynamic loadings. Nevertheless, few studies have been performed to explore the mechanical behavior of single PI, PBO, and aramid III fibers under dynamic loadings.

In this paper, based on the work of Lim et al. [20] and Wang et al. [21], an improved small SHTB is adopted to investigate the dynamic responses of single PBO, PI, and aramid III fibers. The quasi-static tests are also studied as a comparison. Scanning electron microscopy (SEM) is used to characterize the microstructure change after dynamic failure. Moreover, two-parameter Weibull distribution with a strain-rate term is used to describe the strength characteristics.

## 2. Methods and Materials

### 2.1. Test Method and Principle

The traditional SHTB is mainly composed of a projectile, an incident bar, and a transmitting bar. The incident wave and reflected wave signals are collected on the incident bar, and the transmitted wave is measured on the transmitting bar by strain gauges [22]. Based on the three-wave method [23], the strain rate, strain, and stress of a sample are calculated as follows:

(1) $$\dot{\varepsilon }(t)=C_{0}\frac{\varepsilon_{I}-\varepsilon_{R}-\varepsilon_{T}}{l_{s}}$$

(2) $$\varepsilon (t)=C_{0}\int_{0}^{t}\frac{\varepsilon_{I}-\varepsilon_{R}-\varepsilon_{T}}{l_{s}}dt$$

(3) $$\sigma (t)=\frac{A}{2A_{s}}E\left(\varepsilon_{T}+\varepsilon_{I}+\varepsilon_{R}\right)$$

where *C*_0_, *E*, and *A* are the elastic wave velocity, elastic modulus, and cross-sectional area of the bar, respectively; *l_s_* and *A_s_* are the length and cross-sectional area of the specimen, respectively; and $\varepsilon_{I}$, $\varepsilon_{R}$, and $\varepsilon_{T}$ are measured incident, reflected, and transmitted strain, respectively.

In the present study, the diameters of the single fibers were in the range of 10~20 µm. For a traditional SHTB facility, a severe impedance mismatch occurs between the bar (i.e., steel) and the single fiber specimen, resulting in an extremely weak transmitted wave. Therefore, instead of using a transmitted bar, a force sensor with high sensitivity was adopted to gather the force of the single fibers during tests. Contact of the end of the incident bar with the specimen is regarded as a free interface, and the strain rate and strain of the fiber can be obtained by using the incident wave or reflected wave. The stress-strain relationship of the fiber under dynamic tension is calculated as follows:

(4) $$\dot{\varepsilon }(t)=2C_{0}\frac{\varepsilon_{I}}{l_{s}}$$

(5) $$\varepsilon (t)=2C_{0}\int_{0}^{t}\frac{\varepsilon_{I}}{l_{s}}dt$$

(6) $$\sigma (t)=F/A_{s}$$

where *F* is the force collected by the force sensor.

**Figure 1:**
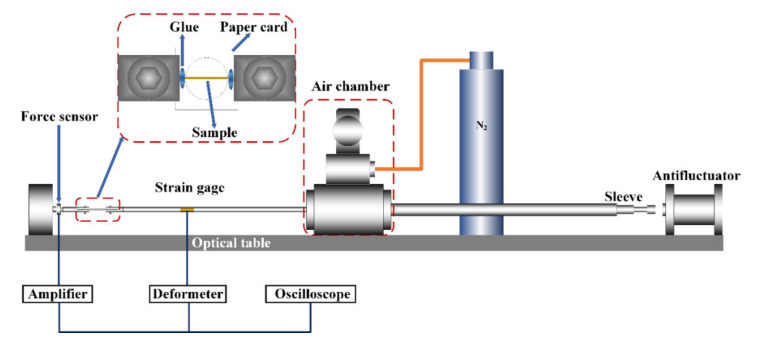
Schematic diagram of the mini-SHTB experiment.

The developed mini-SHTB is shown in Figure 1. A gas gun was used to launch a sleeve-type bullet sleeved on the incident bar. The sleeve bullet hit the mass block fixed at the end of the bar to generate a tensile wave. This propagated along the incident bar and arrived at the sample clamped at the end of the incident bar, causing tensile failure of the sample. The force history was collected by the force sensor. The peak tensile forces were less than 2 N during the tension of each of the single fiber types.

### 2.2. Experimental Samples

**Figure 2:**
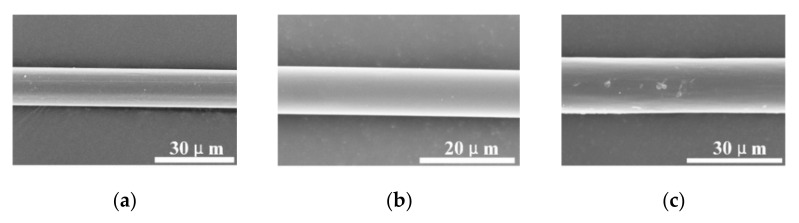
Three fiber categories: (**a**) PBO fiber; (**b**) PI fiber; (**c**) aramid III fiber.

**Figure 3:**
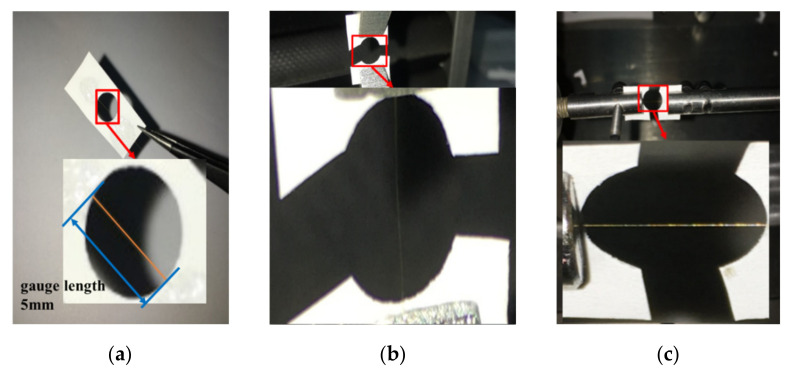
(**a**) Sample shape; (**b**) quasi-static tensile clamping; (**c**) dynamic tensile clamping.

The single PBO, PI, and aramid III fibers used in the present study are shown in Figure 2. The average diameters of the PBO, PI, and aramid III fibers were 14.57, 10.08, and 17.55 µm according to the SEM measurement, respectively. For each strain rate, five and fifteen samples were measured under quasi-static and dynamic tensile tests, respectively, for each fiber category due to the dispersion of the single fibers. The size effect of fibers has been studied intensively; Dyneema SK76 [24], PPTA [25], and Kevlar 129 single fiber [26] all indicate that the longer the fiber length, the lower the strength. The stress wave can be quickly balanced inside the sample to achieve a high strain rate if the fiber length is relatively shorter. According to the studies mentioned above, 5 mm is a common test gauge length that not only provides a high strain rate but also maintains the test accuracy [20,27]. The test parameters are listed in Table 1. In the experiments, the single fiber was glued to the paper card, which had a hole with a diameter of 5 mm, as shown in Figure 3a. Then, the paper card was fixed onto the quasi-static tensile machine, as shown in Figure 3b, or the mini-SHTB, as shown in Figure 3c. Before the experiments, the margin of the paper card was cut carefully to prevent any damage or pre-stretching of the single fibers.

## 3. Results and Discussion

### 3.1. Experimental Results

**Figure 4:**
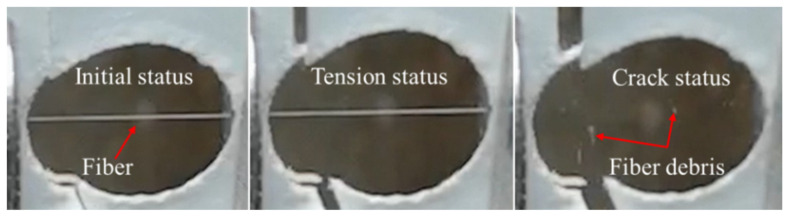
Dynamic tension process.

**Figure 5:**
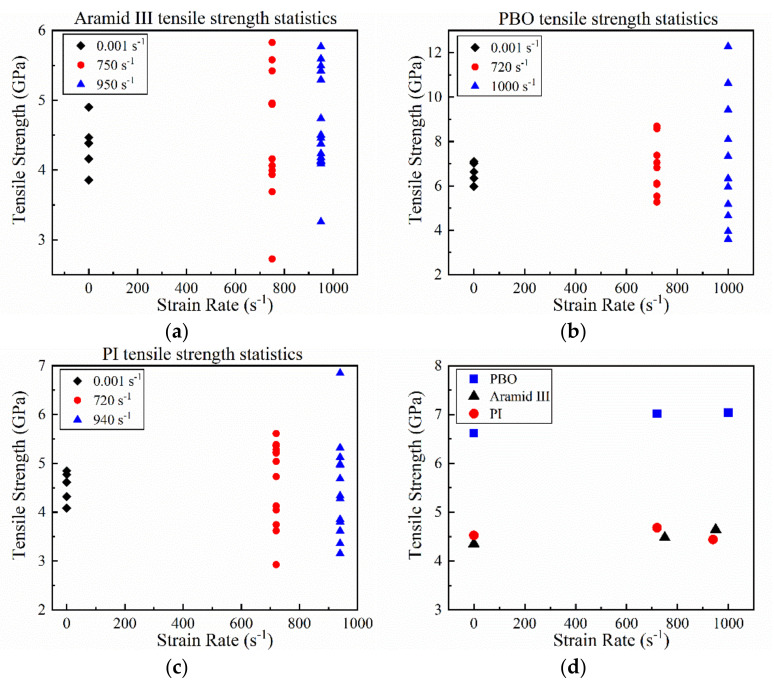
Tensile strengths of (**a**) aramid III fibers at quasi-static, 750 s^−1^, and 950 s^−1^; (**b**) PBO fibers at quasi-static, 720 s^−1^, and 1000 s^−1^; (**c**) PI fibers at quasi-static, 720 s^−1^, and 940 s^−1^; and (**d**) average strength versus strain rate.

The typical deformation and failure behavior of a single fiber under dynamic tension is shown in Figure 4, where the fiber is stretched initially, then fragmented into small debris. Figure 5a–c shows the tensile strength distribution of the single aramid III, PBO, and PI fibers under different strain rates. The related results are listed in Table 2. The strengths of the three single fibers show wide dispersion, which can be ascribed to the internal defects of fibers inevitably introduced during the manufacturing process. Generally, the defect distributions in the same single fibers with large length are close to each other, leading to less scatter in the test results [28]. However, for the single fiber samples with a short length of 5 mm, there is a discrepancy in the defect distribution of the fibers, leading to a large difference at the same loading rates. In addition, the dispersion enlarges at high loading rates. The entanglement, friction, and breakage of polymer chains inside the fibers provide the strength of single fibers. During quasi-static loading, the entangled polymer chains induced by the initial defects can unlock during tension. The friction and breakage of the polymers contribute to the ultimate strength of a single fiber. Therefore, the dispersion of the strength is small under quasi-static tension. However, there is inadequate time for the entanglement of polymer chains in the fibers to unlock during dynamic tension under high strain rates, resulting in a large discrepancy in the ultimate strengths of the fibers under the same loading rates. As shown in Figure 5a, the average failure strength of a single aramid III fiber increases slightly from about 4.35 to 4.64 GPa while increasing the strain rate from 10^−3^ to 950 s^−1^. The average strength of a single PBO fiber increases from 6.61 to 7.04 GPa while increasing the strain rate from 10^−3^ to 1000 s^−1^_,_ as depicted in Figure 5b. As shown in Figure 5c, the average strength of a single PI fiber increases slightly from 4.53 to 4.68 GPa with the increase in strain rate from 10^−3^ to 720 s^−1^, and then decreases to 4.44 GPa with the further increase in strain rate to 940 s^−1^. The comparison of the three fibers is shown in Figure 5d, where the average strength of a single PBO fiber is higher than that of the other two single fibers. This implies the PBO fiber may be preferred in terms of strength when choosing protective materials. However, the mechanical properties of PBO fiber are vulnerable to the irradiation by ultraviolet light, which should be solved in practical applications [29,30].

**Figure 6:**
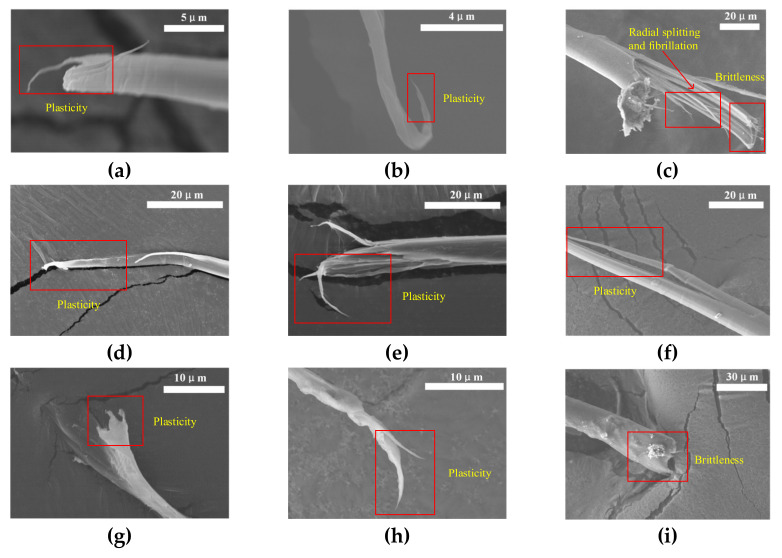
Broken morphologies of PBO fiber with strain rate at (**a**) 0. 001 s^−1^, (**b**) 720 s^−1^, and (**c**) 1000 s^−1^; PI fiber with strain rate at (**d**) 0.001 s^−1^, (**e**) 720 s^−1^, and (**f**) 940 s^−1^; aramid III fiber with strain rate at (**g**) 0. 001 s^−1^, (**h**) 750 s^−1^, and (**i**) 950 s^−1^.

In order to understand the deformation and failure behavior of the single fibers, the morphologies of the samples after testing were observed, as shown in Figure 6. As shown in Figure 6a–c, the single PBO fiber shows obvious plasticity during tension under quasi-static conditions and 720 s^−1^, as evidenced by the obvious stretching and necking near the fractured surface. When increasing the strain rate to 1000 s^−1^, the stretching and necking morphologies disappear, indicating the ductile-to-brittle transition. In addition, a single PBO fiber shows noticeable radial splitting and fibrillation characteristics under 1000 s^−1^, leading to the increase in the failure strength as measured in the experiments, which is consistent with the findings of Xiong et al. [31] and Kitagawa et al. [32]. The fibrillation and radial splitting increases the roughness inside the PBO fiber, resulting in a higher energy absorption capacity. The PI fiber exhibits plastic deformation characteristics during tension at all three strain rates, as evidenced by the obvious stretching and necking near the fracture surface, as shown in Figure 6d–f. No radial splitting or fibrillation characteristics are observed. As shown in Figure 6g–i, similar to the PBO fiber, the aramid III fiber shows obvious plasticity under quasi-static conditions and 720 s^−1^. The fiber transfers from plasticity to brittle failure with increasing the strain rate to 940 s^−1^. However, there is no obvious radial splitting or fibrillation characteristics at a high strain rate, and relative neat fracture morphology is observed at 940 s^−1^. In situ observation will be performed in a future study to further understand the deformation mechanism of single fibers.

### 3.2. Weibull Strength Distribution

For a single fiber, the wide dispersion of strength results from defects in the fiber’s microstructure. The Weibull strength distribution, considering the strain rate effect, is generally used to estimate the cumulative failure probability of single fibers [21,33]

(7) $$P(\sigma )=1-exp\left[-{\dot{\varepsilon }}^{k}{\left(\frac{\sigma }{\sigma_{\dot{\varepsilon }}}\right)}^{m}\right]$$

where $\sigma_{\dot{\varepsilon }}$ is a parameter related to strain rate, *k* is a constant, and *m* denotes the shape parameter of the Weibull distribution. The average fiber strength can be expressed as

(8) $$\bar{\sigma }=D{\dot{\varepsilon }}^{-\frac{k}{m}}$$

where *D* is Γ function related to $\dot{\varepsilon }$ and *m*. Taking the logarithm of both sides of Equation (7),

(9) $$ln\left[ln\left(\frac{1}{1-P(\sigma )}\right)\right]-kln\dot{\varepsilon }=mln\sigma -mln\sigma_{\dot{\varepsilon }}$$

After fitting the experimental data, it yields:

(10) $$P\left(\sigma \right)=\frac{i-0.5}{N}$$

where *N* is the number of test specimens and *i* denotes the number that ranks the strength data from small to large in sequence.

Let $\frac{k}{m}=\xi$, and Equation (9) can be rewritten as

(11) $$ln\left[ln\left(\frac{1}{1-P(\sigma )}\right)\right]=m(\xi ln\dot{\varepsilon }+ln\sigma )-mln\sigma_{\dot{\varepsilon }}$$

**Figure 7:**
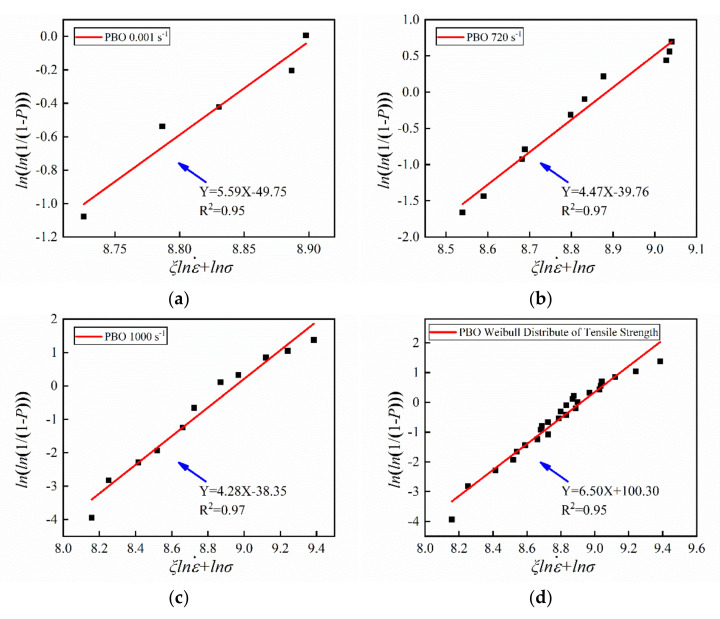
Weibull distribution of single PBO fibers with strain rate at (**a**) 0.001 s^−1^, (**b**) 720 s^−1^, (**c**) 1000 s^−1^, and (**d**) the whole range.

**Figure 8:**
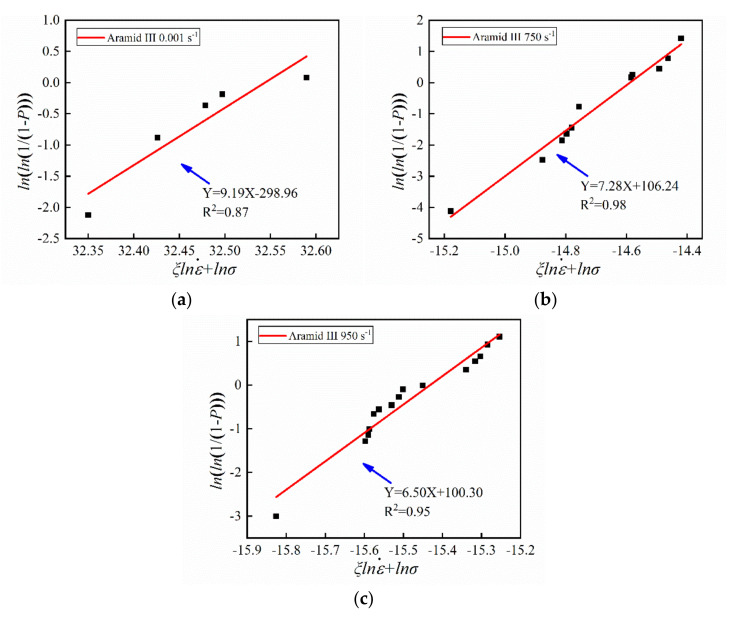
Weibull distribution of single aramid III fibers with strain rate at (**a**) 0.001 s^−1^, (**b**) 750 s^−1^, and (**c**) 950 s^−1^.

By fitting the experimental data, the Weibull distributions of the three fibers were obtained. It is clearly shown in Figure 7 that the PBO fiber is in agreement with the Weibull strength distribution at a different strain rate. In addition, the strength of the PBO fiber also follows the Weibull distribution with the strain rate term. The aramid III fiber also obeys the Weibull strength distribution at each loading rate, as shown in Figure 8. However, it does not conform to the Weibull strength distribution with the strain rate term. For the PI fiber, due to the disagreement with Equation (8), the Weibull distribution could not be fitted. The shape parameter *m* can reflect the dispersibility of the Weibull distribution. The smaller the *m*, the larger the dispersibility. The shape parameters are listed in Table 3, where the dispersibility becomes larger as the strain rate increases for both the PBO and the aramid III fiber. In previous studies [13,34,35], it was generally thought that a single fiber in a fiber bundle conforms to the hypothesis of Weibull distribution. For example, the carbon nanotube fiber measured by Wang et al. [21] conforms to the Weibull distribution with the strain rate term. However, the present study suggests that the hypothesis of the Weibull distribution for single fibers needs to be revisited.

## 4. Conclusions

The quasi-static and dynamic mechanical properties of three high-performance single fibers were measured by a tensile testing machine and an improved mini-SHTB, respectively. The main conclusions are as follows:

(1) The strengths of the single PBO and aramid III fibers are more sensitive to strain rate compared to single PI fibers. In addition, the PBO fiber is superior to the other two fibers in terms of strength and elongation. However, the vulnerability when subjected to ultraviolet light needs to be improved for practical application.
(2) The strength dispersion of single fibers was observed in experiments, and it enlarges with increasing strain rate, which can be ascribed to the effects of the internal defects.
(3) Only the PBO fiber conforms to the Weibull strength distribution with a strain rate term, suggesting that the hypothesis of Weibull distribution for single fibers needs to be revisited.
(4) The single PBO and aramid III fibers exhibit ductile-brittle transition with increasing strain rate, while the PI fiber shows plasticity for all the tested strain rates.

In the experiments, the relationship between the strength and the microstructure evolution is still unclear. In situ observation will be performed in a future study to further understand the deformation mechanism of single fibers.

## Figures and Tables

**Table 1:** Test parameters of the three single fibers.

Fiber	Diameter (μm)	Gauge Length (mm)	Number of Samples (Quasi-Static/Dynamic)	Strain Rate (s^−1^)
PBO	14.57	5	5/15	0.001/720/1000
Aramid III	17.55	5	5/15	0.001/750/950
PI	10.08	5	5/15	0.001/720/940

**Table 2:** Strength (in GPa) of the three fibers at each strain rate.

Type of Fiber	Quasi-Static (0.001 s^−1^)	High Strain Rate Tests	
PBO	6.61 ± 0.47	7.01 ± 1.29 (720 s^−1^)	7.04 ± 2.82 (1000 s^−1^)
Aramid III	4.35 ± 0.39	4.48 ± 0.94 (750 s^−1^)	4.64 ± 0.72 (950 s^−1^)
PI	4.53 ± 0.32	4.68 ± 0.83 (720 s^−1^)	4.44 ± 0.97 (940 s^−1^)

**Table 3:** Shape parameters of the Weibull distribution for different single fibers.

	Strain Rate (s^−1^)	0.001	720/750/720	1000/950/940	Overall
*m*	PBO	5.59	4.47	4.28	4.36
	Aramid III	9.19	7.28	6.50	/
	PI	/	/	/	/

## Data Availability

Data sharing is not applicable to this article.

## Nomenclature

$\dot{\varepsilon }$	Strain rate	$C_{0}$	Wave speed
ε	Strain	$\varepsilon_{R}$	Reflected wave
σ	Tensile strength	$\varepsilon_{T}$	Transmitted wave
$l_{s}$	Sample length	$\varepsilon_{I}$	Incident wave
A	Cross-sectional area of bar	F	Force
$A_{s}$	Cross-sectional area of sample	P	Cumulative failure probability
E	Elastic modulus	*m*	Shape parameter of Weibull distribution
$\bar{\sigma }$	Average tensile strength at respective strain rates	*N*	Number of test specimens
$\sigma_{\dot{\varepsilon }}$	A parameter related to strain rate		
